# ‘Us versus them’: A social identity perspective of internal medicine trainees

**DOI:** 10.1007/s40037-022-00733-9

**Published:** 2022-12-07

**Authors:** Joanne Kerins, Samantha Eve Smith, Victoria Ruth Tallentire

**Affiliations:** 1 Scottish Centre for Simulation and Clinical Human Factors, Larbert, UK; 2 NHS Greater Glasgow and Clyde, Glasgow, UK; 3 NHS Lothian, Edinburgh, UK; 4 NHS Education for Scotland, Edinburgh, UK

**Keywords:** Social identity, Internal medicine, Postgraduate training

## Abstract

**Introduction:**

Silos and group boundaries in the clinical workplace can result in interprofessional conflict which can be a source of anxiety for doctors in training. The social identity perspective (SIP) incorporates theories of social identity and self-categorisation, and may provide a useful lens to understand the socialisation and identity development of doctors. This study aimed to determine if and how the SIP may provide insight into intergroup relations as experienced by internal medicine (IM) trainees in Scotland.

**Methods:**

Interprofessional communication workshops hosted as part of an IM boot camp between August 2020 and March 2021 were audio recorded and transcribed verbatim. Subsequent individual interviews with consenting trainees further explored social identity and intergroup relations. Data analysis employed template analysis and deductive independent coding with the SIP informing the initial coding template and new codes added inductively.

**Results:**

Seventeen workshops, involving 100 trainees, and ten subsequent individual interviews were included. Trainees related to the social identity of an IM doctor and to stereotypes within the workplace. They described intergroup tensions resulting from a perception of differing priorities. They experienced outgroup derogation and the impact of role modelling those in their social group during their identity development.

**Discussion:**

The SIP provides a useful lens to understand the social phenomena at play for IM trainees. It confirms the expectation of conflict between specialties and negative perceptions of outgroups. There is a need to consider the hidden curriculum of socialisation in the workplace during training and the influence of the learning environment on identity development.

**Supplementary Information:**

The online version of this article (10.1007/s40037-022-00733-9) contains supplementary material, which is available to authorized users.

## Introduction

Professional silos in healthcare can lead to social stereotyping [1, 2], conflict [3–6], and suboptimal interprofessional collaboration [3, 7, 8]. Intergroup boundaries are emphasised by the categorisation of healthcare workers into their professional groups [9]. The resultant interprofessional tensions can influence the socialisation and identity development of trainee doctors through role modelling and mimicry [10–13]. In parallel with interprofessional tensions, interspecialty conflict is recognised, for example between internal medicine (IM) and emergency medicine [14], with specialty identities found to be a contributory factor [15]. Workplace tensions and conflict are important; not only are they distressing for the professionals involved [16], they are also associated with an increased propensity for junior doctors to leave training schemes [17], and increased medical error [18, 19]. Although previous work has explored the contextual factors from which tensions can arise, such as roles and resources [12, 14], further work is required to better understand how intergroup boundaries affect the developing physician.

Retention of trainees in IM in the United Kingdom (UK) is a concern, with fewer than half of IM trainees progressing to higher specialty training in recent years [20, 21]. Workplace culture [22] and lack of professional identity [23] have been identified as factors influencing retention of trainees in other contexts. Workplace culture refers to the social constructs that influence acceptable behaviour and social norms [24, 25]. Understanding the workplace culture, as experienced by IM trainees, could inform strategies to aid retention. Interactions with a myriad of colleagues represent a considerable part of an IM trainee’s role and the navigation of such relationships is often learned ‘in the trenches’ of the clinical workplace [11]. Consequently, the clinical learning environment is a site of professional socialisation and identity development, the influence of which remains underexplored [26].

Once trainees embark upon IM training they will further develop and strengthen their professional identity (as a doctor) and their specialty identity (as an IM physician) [27]. Context is important in the professional identity development of IM residents and further research of these sociocultural influences is required [28]. Developing professional social identities can provide a sense of belonging to a group [29] and foster social safety but may also reduce collaboration and increase intergroup conflict. IM trainees are exposed to interprofessional conflicts in the workplace [19] and there have been calls to equip individuals with the ability to reflect on their own and others’ social identities [27]. Using theory from social psychology may allow medical educators to appreciate the causes of interprofessional conflict and shape the way we conceptualise ourselves and others.

### Conceptual framework

Social identity describes the way we perceive ourselves in a social context as a group member [30]. A social group is a collection of people who share the same social identity, for example political affiliation or, within healthcare, specialty or profession [30]. Social identity has been utilised to conceptualise challenges within the clinical workplace [3, 4, 9, 15, 27, 31]. This study uses the social identity perspective (SIP) as a lens to explore intergroup relations. The SIP is an overarching metatheory from social psychology [30] encompassing two main sub-theories: social identity theory [1] and self-categorisation theory [32]. Social identity theory focuses on the role of identity in intergroup conflict whereas self-categorisation theory describes the cognitive processes involved in the categorisation of self and others [33]. We tend to compare our own group (‘ingroup’) with others (‘outgroups’) giving rise to intergroup contrasts, stereotypes and group norms [34]. Within-group differences are often minimised and intergroup differences exaggerated resulting in perceived inferiority and bias. Individuals have various social identities, each more or less salient depending on context [27]. The SIP forms the conceptual backbone for this study, to appreciate the benefits and risks of such social processes to the IM trainee workforce and the influence on their professional identity development.

### Study aim

The aim of this study is to explore how intergroup relations are experienced by IM trainees in the clinical environment in Scotland, using the SIP.

## Methods

### Context

IM training is a three-year training programme for doctors in the UK pursuing a career in medical specialties. For further information on the UK training, see the Electronic Supplementary Materials. In Scotland, a boot camp within the first year of IM training includes an interprofessional communication workshop. This uni-professional session, designed by IM consultants with trainee input, aimed to explore challenges of interprofessional communication. Between August 2020 and March 2021, the workshop was delivered to 124 IM trainees at the Scottish Centre for Simulation and Clinical Human Factors in groups of six with two facilitators. Each group consisted of trainees of different genders from a mix of training regions. The discussion was trainee-led with trainees setting their own agenda and facilitators using open questions to enquire about experiences and prompt reflection on the impact of challenges.

### Data collection

An observational approach was employed, audio recording the workshops in which all participants consented. This approach aimed to gather descriptions of encounters in the clinical workplace, whilst not influencing participants’ dialogue or learning experience [35]. The group dynamic allowed group norms and perspectives on other groups to be explored. JK was a non-participating researcher present at all workshops to record the conversation, take field notes to aid identification of participants from recordings and to immerse herself in the data. Subsequently, all 49 trainees consenting to follow-up interview from the first three workshops were contacted by email and invited to an interview via Microsoft Teams, conducted by JK, to further explore social identity development. A convenience sample of those responding and agreeing to an interview was included. Interviews were semi-structured with probing questions devised relating to the SIP (Interview Guide included in Electronic Supplementary Materials). Interviews continued until the research team deemed that the data gathered provided sufficient information power to address the research aim [36, 37]. Audio recordings of workshops and interviews were anonymised and transcribed verbatim.

### Data analysis

Given the vast body of literature relating to SIP, Hogg et al.’s description of the social identity perspective was chosen for this study as a unified view of underlying concepts [30]. Although social identity theory originated through discussions of large-scale group phenomena, this perspective also provides a useful outlook on smaller groups, such as profession within a workplace [30]. The breadth of research in this area prompted the use of a deductive approach through template analysis rather than an inductive approach. Using Hogg et al.’s components of SIP, as presented in their 2004 paper [30], an initial coding template was developed as summarised, with illustrative examples, in Tab. 1.

**Table 1:** Hogg et al.’s components of the social identity perspective as coding template with illustrative examples [30]

Component of SIP		Description	Illustrative example
*Social identity, collective self and group membership*		‘A social group is a collection of more than two people who have the same social identity—they identify themselves in the same way and have the same definition of who they are, what attributes they have, and how they relate to and differ from specific outgroups’ [30]	Identifying as a Manchester United football fan, as opposed to any other football team supporter
*Social categorisation, prototypes and depersonalisation*		‘People cognitively represent groups in terms of prototypes—fuzzy sets of interrelated attributes that simultaneously capture similarities and structural relationships within groups and differences between the group … you see them through the lens of the prototype—they become depersonalized’ [30]	A group of teenagers being seen as a collective of trouble makers
*Motivation*	*Self-enhancement*	‘People strive to promote or protect the prestige and status of their own group relative to other groups because group evaluation is self-evaluation’ [30]	A political party highlighting the ways they are morally superior than their rival party
	*Uncertainty reduction*	‘People strive to reduce subjective uncertainty about their social world … they like to know who they are and how to behave, and who others are and how they might behave … self-conceptual certainty, renders others’ behaviour predictable and therefore allows one to avoid harm and plan effective action’ [30]	Self-uncertainty in students leading to seeking group membership e.g. joining a radical environmental group
*Social attraction and group cohesion*		‘Social attraction is a function of how much one identifies with the group and how prototypical the other person is—it is positive regard or liking for the prototype as it is embodied by real group members.—the warm feeling of oneness with fellow members’ [30]	Favouritism in recruitment due to religious background with an employer favouring those of the same religious group
*Social comparison*		‘Intergroup social comparisons do not strive toward uniformity and assimilation; instead, they strive to maximize differences between self, as ingroup member, and other, as outgroup member’ [30]	Pupils of one school comparing themselves to another and seeing them as inferior
*Intergroup relations*		‘At the level of intergroup relations, this idea explains why groups compete with each other to be both different and better’ [30]	Citizens of one city competing for the reputation of being a better place to live compared with another city
*Social influence, conformity and group norms*		‘Norms are the source of social influence in groups because they are prescriptive, not merely descriptive. The self-categorization and depersonalization process explains how people conform to or enact group norms’ [30]	Adults in the UK conforming to government advice and wearing masks during the COVID-19 pandemic when in shops or on public transport even after legal restriction ceased

Transcripts were independently analysed using template analysis. In template analysis, a coding framework based on prior research or existing theory is applied to the data deductively, with the option for the initial template to be modified by the data, with new codes added inductively [38]. Due to the vast data gathered, JK performed initial line by line coding of full workshops transcripts to identify relevant sections to answer the research question. All authors then performed focussed coding of the workshop transcripts and line by line coding of the interview transcripts using the coding template described in Tab. 1. The authors coded lines or segments separately that did not fit the a priori template in order to inductively analyse. Disagreements on coding were discussed referring to the coding template in Tab. 1 with final decisions made by JK. The results are therefore JK’s conceptualisation of the framework produced by the interactions between JK, the participants and her co-researchers.

### Reflexivity

In this constructivist study, ideas were co-constructed between participants, facilitators and researchers. We recognised that our prior clinical and educational experiences, particularly of hospital-based professional relationships, would influence the findings. JK is an acute IM registrar and medical educator with significant medical education research experience. SS is a general practitioner and medical educator with a medical education doctoral degree. VT is an acute IM consultant and medical educator with a medical education doctoral degree. The combination aimed to provide a range of perspectives on the data. The interprofessional workshop facilitators are medical consultants and educationalists and their own experiences of hospital-based professional relationships will have influenced their interactions with trainees during the workshops. Given the observational aspect of this part of the study they were not aware of the research question being examined and so were not influenced by this in their discussions.

## Results

Seventeen workshops, each two hours in duration, involving 100 trainees, were included in the study. Ten trainees took part in the subsequent interview process including seven identifying as female and three identifying as male. Individually interviewed trainees were aged 24–35. Interviews lasted between 18 and 35 min (mean 28 min). Quotes from workshops are indicated with ‘W’ after trainee code, and quotes from interviews indicated with ‘I’. The results are summarised in Tab. 2 including modifications to the template in this context.

**Table 2:** Components of SIP with subtheme modifications in the context of IM trainees with example quotes

Component of SIP from IM trainees’ perspective		Example quote
Social identity, collective self and group membership		*‘We think of ourselves as medics- you’ve got the GPs [General Practitioners], the medics, the surgeons … we do categorise ourselves into those areas’* (T104I)
Social categorisation, prototypes and depersonalisation		*‘There’s certain specialties … I mean, it’s the same in every area, have a reputation … there are some where you know it’s going to feel … might be a bit more challenging on the phone in terms of accepting referrals’* (T9W)
Motivation	Self-enhancement	*‘You can’t bad-mouth your own, so you’ve got to bad-mouth someone’* (T6W)
	Uncertainty reduction	*‘I wonder whether actually sometimes for me now it is more the expectation of tribalism that then colours the way that I approach the conversation, and then if you start off on that foot then you set the tone of the conversation’ *(T11I)
Social attraction and group cohesion		*‘I think usually within your own team it’s less of a problem generally because you feel part of the team … there’s been a bit more communication through the day about things’ *(T9W)
Social comparison	*Differing priorities*	*‘Their [the surgeons] priority is often the patients that they can go and do a definitive thing to, do an operation on, fix a problem … each specialty has its own hierarchy of problems and they don’t often mesh’* (T3W)
Intergroup relations	*Tension*	*‘I’ve never been shouted at by my specialties from the hospital but I’ve been shouted at by ED [Emergency Department] … when I was working in ED I wasn’t shouted at’ (T51W)*
Social influence, conformity and group norms	*Outgroup derogation*	*‘… and some of that becomes learned, there’s this thing that everybody knows that talking to neurosurgery or talking to the bed manager is a nightmare … it becomes this sort of banter thing where you just use a particular department as a butt of a joke’* (T18W)
	*Hierarchy*	*‘A nurse in charge didn’t care when a consultant wore his smart watch but when I wore one he did care … it seems like there is a different rule’ *(T87W)

### Social identity, collective self and group membership

The social identity trainees expressed in this study was that of being a doctor: *‘I just feel like, as doctors, we quite like being able to fix things*’ (T6W). More specifically, they identified as a ‘*medic*’ in the hospital, a term used to refer to IM in the UK, as opposed to other specialties. IM trainees referred to themselves as ‘*we’* when describing IM as a specialty, suggesting they situated themselves firmly within this group.

Most trainees declared that they ‘*feel like a medic*’ (T3I). This was due to ‘*the fact that I am just doing medicine now … I am studying for my MRCP *[Membership of the Royal College of Physicians] *… definitely this is me now … it is all about the medicine side of things’ *(T3I) as opposed to during foundation training when they rotated through various specialties. Some felt that from an earlier stage *they ‘always had a medical hat on’ *(T103I) and during their foundation training was ‘*when I started to think of myself as a more medically minded person’* (T104I). This referred to having ‘*a more rounded view*’ (T17W) and being able to ‘*provide a better care for multiple systems*’ (T17W). For others the sense of specialty identity development took longer:

*“It is taking me quite a long time to identify as a medic … it has taken me a long time to actually feel this switch but, I think, slowly I am. The more I go into clinics, the more I am dictating, the more I am managing more unwell patients *(T11I).”

They perceived their stage of training as an ‘*awkward phase*’ (T21W) and a bit of a ‘*grey area*’ (T28W) as they progress from junior to senior which they saw as a challenge for others to recognise their role. They sometimes described themselves not by what they are, but what they are not: ‘*I’m an IMT *[IM trainee]*, so that’s like a middle grade … we’re in that stage where we’re not registrars, but we’re not foundation *[more junior doctors]’ (T25W).

**Fig. 1:**
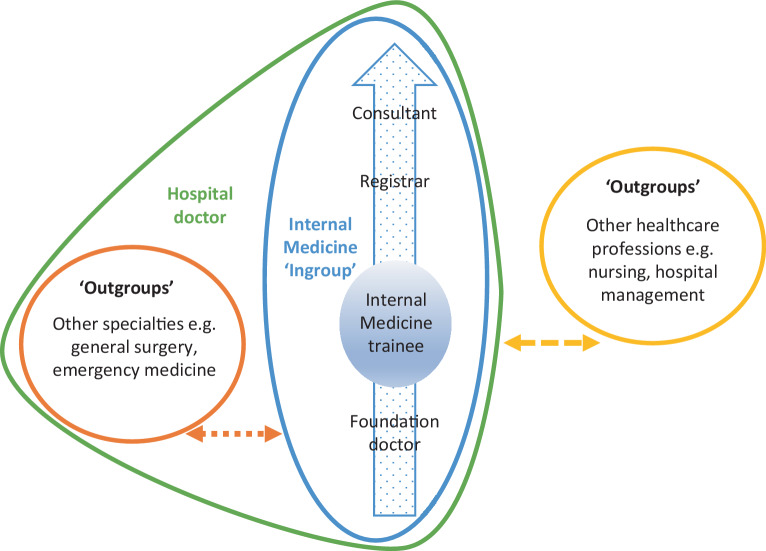
Diagrammatic representation of IM trainees’ social identity in the workplace and interactions with outgroups

Fig. 1 displays a diagrammatic representation of the social identity they described.

### Social categorisation, prototypes and depersonalisation

IM trainees recognised the existence of stereotypes within the workplace and that ‘*certain specialties have a reputation*’ (T9W). Surgical specialties always ‘*busy in theatre*’ (T9W), nursing staff were ‘*pedantic*’ (T40W) regarding prescriptions or protocols and bed managers were preoccupied with ‘*targets being missed in A&E* [Accident and Emergency]’ (T42W). Although some aspects of the stereotypes were regarded in a jovial way, it was recognised that such preconceptions can have significant negative impacts in the workplace:

*“A lot of it is obviously in jest, there are a lot of jokes like, orthopaedic surgeons can’t read an ECG, that are just light-hearted, but I do feel like sometimes it can be a slippery slope … I think it is important as juniors to try defusing that a bit and say everybody has their own different skills. Everyone has a different skill set; we can’t do what they do* (T104I).”

They also reflected on how others may perceive their group:

*“They *[obstetrics trainees] *were chatting to me about how frustrating it is for them, because they get so much grief when they try and get a medical opinion. They were saying “medics just think they’re such martyrs and they’re always so busy” and I thought “is that what we’re like?” (*T28W).”

This interaction prompted reflection on how they may be perceived by others and a realisation that ‘*it’s really easy to get sucked into that cycle of *“*woe be me”*’ (T28W) especially when busy. They felt a need to try to ‘*catch yourself*’ (T28W) when feeling this way and remember everyone is busy but that this self-pitying tendency was often the default attitude: ‘*I think that’s the culture that medics are probably more likely to slide into when they’re stressed’ *(T28W).

Another perception they had heard from other specialties was that ‘*medics think they are so much better than everyone else, they think they know everything*’ (T104I). They could understand why others thought this as there was a habit when receiving referrals, from general practitioners in particular, to think ‘*I cannot believe they referred this to me, we would never do that*’ (T104I). However, some perceived IM to have less of a stereotype compared to other specialties: ‘*I think there’s less of a stereotype of a medic than there are of other specialties … I think the majority of stereotypes are about other specialties, which is unfortunate. I think we maybe are quite lucky’ *(T12I).

### Motivation

#### Self-enhancement:

The perception of IM not having a stereotype could be considered self-enhancement in itself. There was also evidence of self-enhancement with the IM trainees describing themselves as ‘*a bit more holistic*’ (T41W) than other specialties. They stated that ‘*in medicine we’re not obstructive physicians, we’re really caring as well*’ (T97W) when comparing themselves with surgical specialties. They described their specialty favourably compared with surgery: ‘*I would prefer, if I were in hospital, to be on a medical ward.*’ (T41W). This self-enhancement inevitably resulted in social comparison and perceived inferiority of outgroups.

#### Uncertainty reduction:

Trainees regarded the behaviours of other specialists somewhat predictable and found themselves ‘*going into these conversations with a preconception that it’s going to go awfully’* (T6W). However, rather than finding comfort in uncertainty reduction, IM trainees found these attitudes unhelpful and were keen to try to avoid such preconceptions. There appeared to be cognitive dissonance around the depersonalisation of another group.

### Social attraction and group cohesion

There was evidence of ingroup cohesion, particularly if junior colleagues suggested that they were interested in pursuing an outgroup specialty:

*“When we talk about them, as we’re medics, we don’t just tease them about the surgical specialty, but we give them a persona that isn’t a very flattering one, whereas we think of ourselves as lovely and kind and nicey-nice *(T58W).”

Trainees reflected on group cohesion during time spent in neurosurgery during their foundation training when ‘*they were absolutely lovely to us and then they’d go on and off the phone being horrific while they’re sitting having a nice conversation with you*’ (T23W). This is exemplified in Fig. 1 where foundation doctors are part of the ingroup while rotating in that specialty.

Within the last three components of the SIP below, there were specific subthemes identified within this context as modifications to the initial coding template, highlighted in bold and illustrated in Tab. 2.

### Social comparison

Trainees expressed perceived differences with other groups such as nurses being *‘quite rigid about when they take their breaks*’ (T55W) or about following protocols. However, they recognised that nursing staff ‘*get a lot more trouble when things go wrong*’ (T95W) and that ‘*the nursing culture’s a lot more punitive than the medical culture*’ (T24I).

#### Differing priorities:

The salient reason for perceived intergroup contrast was that of *differing priorities*. They reflected ‘*with differing priorities sometimes it’s number ten on your list, whereas it’s top on their list*’ (T6W). This was pertinent regarding interactions with surgical specialties and emergency medicine:

*“In A&E your priority is to see someone’s immediate problems and stabilise them … whereas I guess in medicine there’s more longer-term things and a bit more problem solving, you need more time to look at things. So, I guess you can get frustrated that maybe in A&E, x, y and z haven’t been done … I think that’s the difference in priorities* (T52W).”

They considered that this may be because in A&E they have ‘*the four-hour thing *[government target]* hanging over your head’ *(T8W). The IM trainees perceived contrasting priorities with the hospital bed management team: ‘*Patients out, out, out, no matter what the situation is, completely the opposite to us’ *(T41W).

This clash of priorities could sometimes ‘*come across as a fight between what we’re trying to achieve’ *(T40W). They reflected on their experience in surgery in foundation training when they felt their ‘*sole goal in life was to bounce referrals and get patients out of hospital*’ (T3W). Trainees’ perception of surgical priorities was that, ‘*if they’re not going to operate, then it’s not their issue*’ (T39W).

### Intergroup relations

#### Tension:

There was evidence of *tension* between doctors and nurses with inflammatory language such as ‘*that’s a Datixable offence’ *(T51W), referring to the process of incident reporting as a threat. Intergroup relations often resulted in *tension* or conflicts that trainees found distressing: ‘*You get really scarred by these sorts of conversations … you definitely learn from it, it’s not a fun way to learn, but you do learn’ *(T20W).

This related to conversations about transferring care of a patient to another specialty or requesting a review or advice. Trainees learned when making these requests to ensure they ‘*know everything or have everything in front of you*’ (T20W) to avoid being berated.

Telephone calls were particularly challenging with trainees ‘*in tears about the referrals*’ (T19W): ‘*I got in a conflict with another specialty registrar … I could tell they were probably having a bad day … he actually hung up on me … the conversation was about an inappropriate job to the F1 [Foundation doctor] … and I was properly upset’* (T57W).

This kind of interaction was generally accepted as ‘*just one of those things*’ (T19W) in the work environment. They felt ‘*there isn’t really ever a good reason to make someone feel bad about themselves*’ (T20W) but would ‘*try and remember that it’s not personal*’ (T20W).

### Social influence, conformity and group norms

#### Outgroup derogation:

Trainees described a group norm of *outgroup derogation*, not specific to one group but pervasive throughout the hospital: ‘*All the specialties I’ve been in, they’ll end up doing this bashing … it’s a therapeutic bashing of the other group’ *(T73W).

The therapeutic aspect was ‘*an element of bonding through shared frustration*’ (T4I). It appeared more acceptable to vent frustration at a group rather than an individual: ‘*I’ve vented frustration, I don’t do [it] towards a specific person, but a group of people I tend to just say, you know, “bloody A&E”, that kind of thing’ *(T4I).

Trainees were exposed to negative perceptions of other groups with ‘*an us-versus-them’ *(T40I) mentality. Being geographically distinct made it easier to depersonalise others: ‘*If I hear a voice obstructing me, rather than see you, I’m going to dehumanise you to an extent’ *(T16W).

#### Hierarchy:

Trainees referred to the medical *hierarchy* as an example of prescriptive norms affecting behaviour. They found that their grade heavily influenced the response from other professional groups: ‘*You request a scan and it’s rejected and then the registrar calls and they say “ok it will be done today”’ *(T90W).

Hierarchy was relevant to their social identity: ‘A*s a middle grade you are sort of a teenager of a doctor, you’re basically a baby still, you are two levels below these consultants, that becomes very relevant when they *[other specialties]* don’t know you’* (T95W).

Hierarchy was an influential factor both within their group and when interacting with other groups as displayed in Fig. 1.

Overall, the SIP exhibited how trainees see themselves and others in the workplace. Their social identity, particularly as part of IM as a specialty, was informed through social comparison with other specialties. There was tension between groups and a tendency for outgroup derogation which, although it could lead to ingroup cohesion, trainees perceived as a detrimental habit.

## Discussion

Social identities are a concept that IM trainees can relate to, as well as stereotypes and contrasts with other groups which can be sources of conflict. Their current experience provides a springboard for consideration of strategies to improve the learning environment they are exposed to. The SIP facilitates an insightful conceptualisation of the clinical workplace and the silos that persist in hospital medicine in the UK.

IM trainees identified as ‘medics’ in the hospital, a term used to describe IM in the UK, and conceptualised this group in a positive light. Differing priorities was a source of tension with other groups in the workplace. This aligns with the concept of intergroup conflict theory and the perception of incompatible goals [1]. This perceived incompatibility was common and the expectation of conflict impacted how they approached interactions. Tensions regarding communication, particularly over the phone, and the resulting atmosphere created was reflected on negatively. There are positive aspects to challenging interactions by contributing to physicians’ workplace learning through motivating them to improve [39]. Although IM trainees did learn from difficult interactions, it was not enjoyable and they felt ‘scarred by them.’ Strategies to counter the challenges highlighted include promoting perspective taking and showing appreciation of others through inclusive language [31, 40]. A shift to a cross-cutting identity, spanning across specialty groups, with the concept of ‘the patient’s team’ has been advocated to dispel some of the allegiance to one’s own specialty from which conflict can arise [15].

Trainees recognised intergroup silos with outgroup derogation and potential benefits of a ‘therapeutic bashing’ of other groups. This resonates with the concept of intergroup conflict creating group cohesion as Sumner referred to in 1906: “the exigencies of war with outsiders are what makes peace inside” [41]. Although there was some ‘shared bonding’ through this process, trainees felt uncomfortable with outgroup derogation. There appears to be a double-edged sword to developing social identities in the workplace. It is important to consider this cognitive dissonance that trainees may experience during their professional identity development. Should trainees conform to the group norms of outgroup derogation or try to change the narrative which could influence their predecessors and the workplace culture going forward? This study exhibits an appetite for the latter which medical educators should heed and try to foster. IM trainees recognised the influence that group behaviour can have on their identity development referring to being moulded by their social groups to some extent. Echoing discourse patterns of one’s group has also been highlighted in the professional identity development of novices in the operating room [13]. Not only could this result in role modelling disrespectful behaviour, it could unduly influence junior trainees’ career decisions by referring to other specialities in a negative manner. There must be more productive ways of fostering belonging within specialties than resorting to criticising others.

### Implications for practice

This application of the SIP could be utilised by faculty and trainees to understand and reflect on their perceptions and biases. The findings provide a theoretical grounding for the interprofessional workshop going forward and echoes calls for an empathic workplace culture [42]. Medical educators could consider similar educational interventions informed by the SIP as an opportunity to expose the hidden curriculum of socialisation and professional identity development during training.

### Strengths, limitations and future work

This national, multicentre study informs understanding of IM trainees’ social identities in the clinical workplace. The triangulation of data from observation of workshops and individual interviews allowed an in-depth exploration of social identity and intergroup relations. The observational nature of the workshops did not afford the opportunity for probing questions which an interview-only study could have provided. The perspectives are from IM trainees and future work including other specialties’ perspectives should be considered. We have multiple social identities, for example being a parent, that interact and intersect [43]. Intersectionality, the ways in which social identities interact is an important avenue for ongoing research [27].

There are strengths and limitations to the group setting of the workshop which could prevent individuals vocalising sensitive reflections but may also incite the admission of similar experiences. The consultant faculty may have influenced trainees’ willingness to disclose experiences and preconceptions. Trainees volunteering to be interviewed may hold particularly strong views. JK is an IM registrar which may have affected the way in which trainees described their social identity in interviews. It was reiterated that trainee reflections would be deidentified.

The use of a framework from social psychology and the recognition of similar problems across the hospital increases the potential transferability of the results. This study aimed to focus on the SIP and therefore potentially overlooks other aspects of intergroup dynamics. Future work could focus on identifying strategies to target the phenomena outlined. We should consider how social identities develop throughout training and how educators may play a role.

## Conclusions

This study explores the workplace experiences of IM trainees in Scotland using the SIP. It confirms the expectation of conflict between specialties and exposure to negative perceptions of outgroups. It reinforces an opportunity to use theory from social psychology as an educational tool in exposing this hidden curriculum. The findings should be of interest to medical educators keen to improve the learning environment for trainees developing their social identity in the workplace.

## Supplementary Information

Context of UK training pathway

Interview guide
