# Supplementary material for: ‘Us versus them’: A social identity perspective of internal medicine trainees
Source: Perspect Med Educ. 2022 Dec 7;11(6):341–9. doi: 10.1007/s40037-022-00733-9 (PMC9734785; doi:10.1007/s40037-022-00733-9)
Supplement: Supplementary file 1 — Context of UK training pathway [file 40037_2022_733_MOESM1_ESM.docx]

**Electronic Supplementary Material**

Context of UK training pathway

To enter IM training doctors must have completed a medical degree and a two-year foundation programme where they rotate through various specialities. Following this, doctors in the UK tend to diverge into different training pathways, for example general practice or surgical specialties. It is also possible to start IM training by proving competencies via an alternative route, particularly for those who have trained in other countries. It is also not uncommon for doctors to have had further clinical experience between the foundation programme and IM training. This process is displayed in Figure A below:

Break from training e.g. clinical fellow post or clinical experience abroad

Other specialist training (e.g. surgery, anaesthetics, general practice)

Figure A: Traditional process of progression from medical school to IM training in the UK
