# Supplementary material for: ‘Us versus them’: A social identity perspective of internal medicine trainees
Source: Perspect Med Educ. 2022 Dec 7;11(6):341–9. doi: 10.1007/s40037-022-00733-9 (PMC9734785; doi:10.1007/s40037-022-00733-9)
Supplement: Supplementary file 2 — Interview guide [file 40037_2022_733_MOESM2_ESM.docx]

**Interview guide**

*Social identity, collective self and group membership*

1. To what extent do you identify as a medical doctor?

- If not- when do you think you will?

*Social attraction and group cohesion*

1. Do you think there is a stereotype of a physician in training/medical trainee?

- Do you think you fit that description?

*Intergroup relations*

In the interprofessional workshop there was some discussion about challenging interprofessional communication, between specialties, between doctors and nurses etc

1. Is this something you’ve experienced?

- If so, in what ways?

*Social categorisation, prototypes and depersonalisation*

1. Do you think we tend to see other groups in a stereotypical way?

*Social comparison*

1. If so, in what ways?

*Social influence, conformity and group norms*

In some of the workshops there was discussion around ‘bad-mouthing’ other specialties

1. Is this something you’ve experienced?
2. Why do you think that happens?
